# Histone methylation changes are required for life cycle progression in the human parasite *Schistosoma mansoni*

**DOI:** 10.1371/journal.ppat.1007066

**Published:** 2018-05-21

**Authors:** David Roquis, Aaron Taudt, Kathrin K. Geyer, Gilda Padalino, Karl F. Hoffmann, Nancy Holroyd, Matt Berriman, Benoît Aliaga, Cristian Chaparro, Christoph Grunau, Ronaldo de Carvalho Augusto

**Affiliations:** 1 Technical University of Munich, Department of Plant Sciences, Freising, Germany; 2 European Institute for the Biology of Aging, University of Groningen, University Medical Centre Groningen, AV Groningen, The Netherlands; 3 Institute of Biological, Environmental, and Rural Sciences (IBERS), Aberystwyth University, Penglais, Aberystwyth, Ceredigion, United Kingdom; 4 Wellcome Sanger Institute, Wellcome Genome Campus, Hinxton, Cambridgeshire, United Kingdom; 5 Univ. Perpignan Via Domitia, IHPE UMR 5244, CNRS, IFREMER, Univ. Montpellier, Perpignan, France; George Washington University, UNITED STATES

## Abstract

Epigenetic mechanisms and chromatin structure play an important role in development. Their impact is therefore expected to be strong in parasites with complex life cycles and multiple, strikingly different, developmental stages, *i*.*e*. developmental plasticity. Some studies have already described how the chromatin structure, through histone modifications, varies from a developmental stage to another in a few unicellular parasites. While H3K4me3 profiles remain relatively constant, H3K27 trimethylation and bivalent methylation show strong variation. Inhibitors (A366 and GSK343) of H3K27 histone methyltransferase activity in *S*. *mansoni* efficiently blocked miracidium to sporocyst transition indicating that H3K27 trimethylation is required for life cycle progression. As *S*. *mansoni* is a multicellular parasite that significantly affects both the health and economy of endemic areas, a better understanding of fluke developmental processes within the definitive host will likely highlight novel disease control strategies. Towards this goal, we also studied H4K20me1 in female cercariae and adults. In particular, we found that bivalent trimethylation of H3K4 and H3K27 at the transcription start site of genes is a landmark of the cercarial stage. In cercariae, H3K27me3 presence and strong enrichment in H4K20me1 over long regions (10–100 kb) is associated with development related genes. Here, we provide a broad overview of the chromatin structure of a metazoan parasite throughout its most important lifecycle stages. The five developmental stages studied here present distinct chromatin structures, indicating that histone methylation plays an important role during development. Hence, components of the histone methylation (and demethylation) machinery may provide suitable Schistosomiasis control targets.

## Author summary

*Schistosoma mansoni* is a parasitic flatworm and causative agent of intestinal schistosomiasis, a neglected tropical disease affecting 67 million people worldwide. The parasite has a complex life cycle involving two consecutive obligate hosts (a poikilotherm snail and a homeotherm mammal) and two transitions between these hosts as free-swimming larvae. Here, we show that the chromatin structure of five different developmental stages is characterized by specific changes in chemical modifications of histones, basic proteins that are closely associated with DNA (trimethylation of lysines 4 and 27 and histone H3, and monomethylation of lysine 20 on histone H4). These modifications occur around protein coding genes as well as within repetitive genomic elements. A functional role for histone methylation during schistosome development was elucidated by the use of epi-drugs targeting G9a/GLP and EZH2 histone methyltransferase orthologs in *S*. *mansoni*. Our results indicate that histone methylation plays an important role during schistosome development and suggest that the enzymes responsible for maintaining these chromatin modifications are suitable targets for anti-schistosomal drugs.

## Introduction

Parasites often display complex life cycles that involve several hosts and multiple, phenotypically distinct, developmental stages. This is the case for many human endoparasites, which cause deadly diseases including malaria (*Plasmodium falciparum*), leishmaniasis (*Leishmania spp*.), cryptosporidiosis (*Cryptosporidium spp*.), Chagas disease (*Trypanosoma cruzi*) and schistosomiasis (*Schistosoma mansoni*, *haematobium* or *japonicum*) [1]. Several recent studies have shown that epigenetic mechanisms play an important role in the capacity of these parasites to quickly adapt to a different environment and/or host, to alter their phenotypes at several key points of their life cycles and to evade host defenses [2–7]. In this manuscript, the term “epigenetics” will be used in the 'molecular' sense *i*.*e*. any change in chromatin structure [8]. A better understanding of the proximal epigenetic mechanisms underlying parasite development will help establish novel strategies for treatment or prevention [7]. While chromatin structure changes between life cycle stages were previously documented in a unicellular human endoparasite (i.e. *P*. *falciparum*), a similar approach has yet to be taken for a multicellular human endoparasite [7]. This is likely due to the fact that multicellular parasites present a challenge in term of epigenetic analyses, because they are composed of various tissues with each potentially containing a distinct epigenetic signature.

If developmental epigenetic changes are detectable in multicellular parasites, they can either reflect global changes in epialleles or changes in the relative number of cells with different chromatin structures within the individual. This is similar to a situation in which epialleles are investigated in a population, and we will therefore use epiallele frequencies as a proxy for chromatin structure profiles. Here, we define “epiallele” as a variation of the same genetic locus that differs in their chromatin structure but not in their DNA sequence or genomic position. Epiallele frequencies represented along DNA sequences will be referred to hereafter as chromatin landscapes. Average profiles over genetic units such as genes will be called chromatin profiles or signatures. The present work investigated how epialleles in the chromatin landscape change during the development of the human parasite *Schistosoma mansoni*.

**Fig 1:**
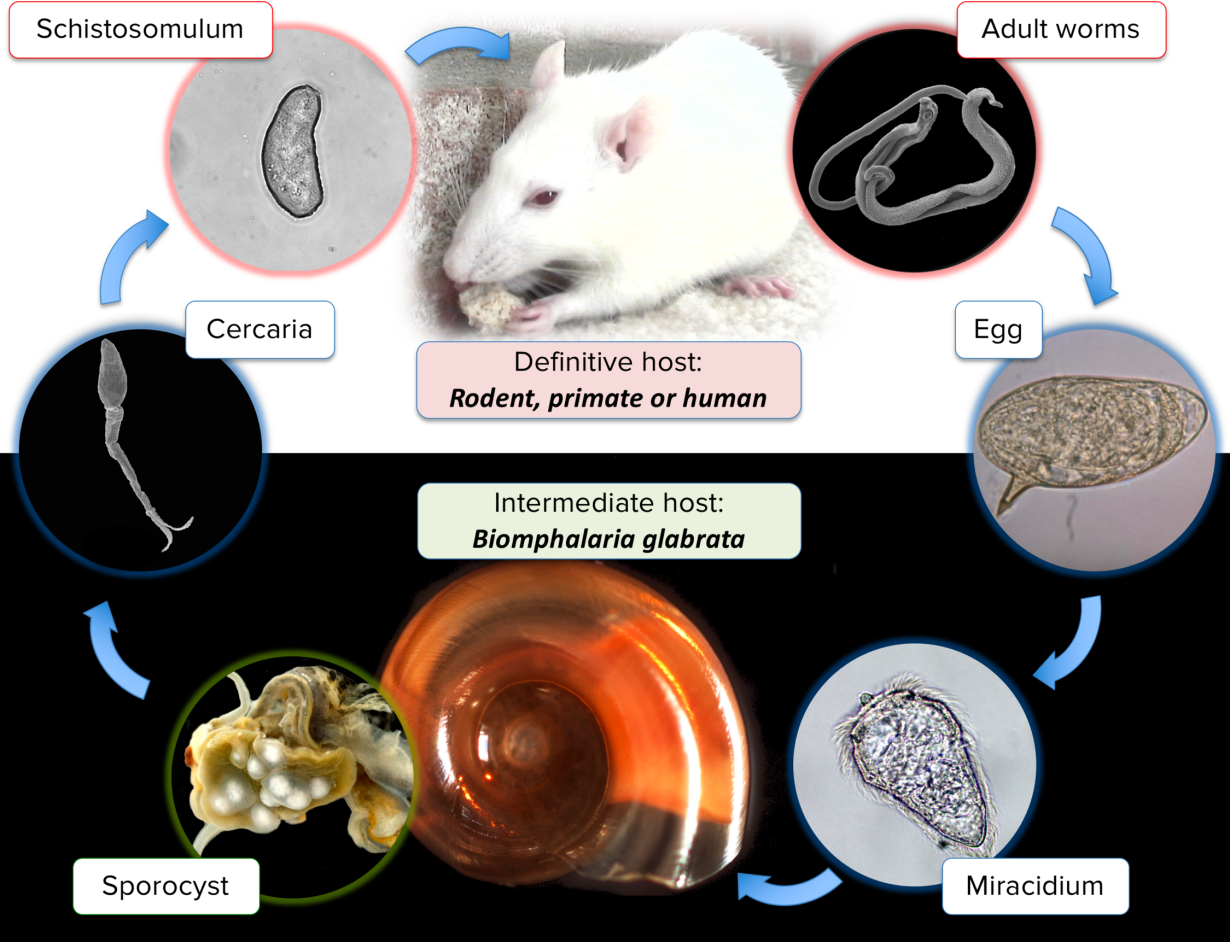
Life cycle of the human parasite *Schistosoma mansoni* including the five developmental stages presented in this work. The life cycle starts when eggs are in contact with freshwater and release a free-swimming larva, the miracidium. Miracidia seek out an intermediate host, a freshwater snail of the *Biomphalaria* genus, penetrate the tegument and transform into primary sporocysts. Sporocysts multiply asexually for approximately ten days and then mature into secondary sporocysts, which generate hundreds of cercariae, a second type of free-swimming larva. Cercariae actively seek a definitive mammalian host (rodent, primate or human) and penetrate the dermis of the host, reaching the vascular system. Schistosomula follow a complex maturation process, ultimately leading to adult worms. Male and female worms form pairs and migrate toward mesenteric veins, where a single female can lay approximately one-hundred eggs per day.

*S*. *mansoni* is a parasitic platyhelminth (flatworm) responsible for intestinal schistosomiasis (or bilharzia), a neglected tropical disease present in Africa, Caribbean, Middle East, Brazil, Venezuela and Suriname [9]. The parasite has a complex life cycle involving two consecutive hosts (a freshwater snail and a mammal) and six major developmental stages (**Fig 1**). Eggs released via the feces of the definitive vertebrate host give rise to a free-swimming miracidium larva, by contact with freshwater. Miracidia seek out an intermediate host, a freshwater snail of the *Biomphalaria* genus [10], penetrate the tegument and transform into primary (Sp1, or mother) sporocysts. For approximately three to five weeks, sporocysts multiply asexually and mature into secondary (Sp2, or daughter) sporocysts and then generates hundreds or thousands of cercariae, a second type of free-swimming larva, per day. Cercariae actively seek a definitive mammalian host (rodent, primate or human [11]), where they penetrate the dermis and mature into schistosomula before reaching the vascular system. Schistosomula follow a complex maturation process, ultimately leading to adult worms. The adult stage is dimorphic with a ZZ sex chromosome pair found in males and a ZW sex chromosome pair found in females. Schistosome development is thus characterized by strong developmental plasticity as illustrated by diverse morphologies, sizes, structures and organs (**Fig 1**). This phenotypic variability has also been found to respond to different environmental stresses (water quality [12], intermediate [13], and definitive hosts’ bodies [14], and drugs [15],). In a previous study, we examined how chromatin structure, represented by three histone modifications, changed during the transition from cercariae to adult stages [16]. We observed that both stages contained a characteristic chromatin signature exemplified by the presence of both, trimethylation on lysine 4 of histone H3 (H3K4me3) and trimethylation on lysine 27 of histone H3 (H3K27me3) marks at the 5’ region of a subset of genes. Our data indicated that, in cercariae, H3K4me3 and H3K27me3 can be present simultaneously (bivalent methylation). Such bivalent marks are likely to be associated with a poised transcriptional state, since we did not find active transcription in cercariae [16]. Transcription of these genes was resumed in the schistosomula stage, when the repressive H3K27me3 mark was removed.

In the present study, we investigate how the chromatin signature changes over the major developmental stages of the parasite: miracidia, primary sporocysts (Sp1), cercariae, schistosomula and adults, and whether other marks might be essential for the human-infecting cercariae to allow their development into adults upon skin penetration. We show here that the frequency of histone methylation marks starts at low levels in miracidia and increases progressively until the adult stage, where sexual reproduction occurs. We also found that the *Schistosoma* life cycle is characterized by two waves of H3K27 methylation/demethylation around the transcription start site (TSS) of genes; one wave with a maximum of H3K27 trimethylation in Sp1 and another wave of H3K27 trimethylation in adults. Furthermore, bivalent H3K4me3 and H3K27me3 methylation (at the same locus) starts in sporocysts and continues until the adult stage, with the highest frequency observed at transcription start sites in cercariae. The distinct chromatin profile over the life cycle indicate that histone methylation plays an important role during development and we have demonstrated that inhibitors of histone methyltransferase orthologs in *S*. *mansoni* arrests miracidium to sporocyst transition. As Picard et al. [17] has previously shown that H3K27me3 demonstrates sex-specific profiles already at the cercarial stage, we therefore decided to further investigate the female epigenome during cercariae to adult transition. Here we included H4K20me1 [monomethylation on lysine 20 of histone H4]). We observed 1,100 peaks that differed for the three histone marks when human infecting female cercariae develop into female adults. The majority of these differences (946) was characterized by a differential enrichment in female cercariae (*i*.*e*. presence of a peak or a stronger peak in cercariae, when compared to adults). Also, we discovered ‘ranges’ spanning 10 to 100 kb, where either H3K27me3 frequency, H4K20me1 frequency, or both, were much stronger (differentially enriched) in female cercariae, 98.6% of the time. A gene ontology overrepresentation analysis showed that these ranges with enrichments in H4K20me1 (either alone or in conjunction with H3K27me3) comprise many genes related to development and regulation of transcription.

## Materials and methods

### Ethics statement

Housing, feeding and animal care at Perpignan followed the national ethical standards established in the writ of 1 February 2013 (NOR: AGRG1238753A) setting the conditions for approval, planning and operation of establishments, breeders and suppliers of animals used for scientific purposes and controls. The French *Ministère de l’Agriculture et de la Pêche* and French *Ministère de l’Éducation Nationale de la Recherche et de la Technologie* provided permit A66040 to our laboratory for experiments on animals and certificate for animal experimentation (authorization 007083, decree 87–848) for the experimenters. All mouse procedures leading to the isolation of miracidia and transformation into sporocysts performed at Aberystwyth University (AU) adhered to the United Kingdom Home Office Animals (Scientific Procedures) Act of 1986 (project license PPL 40/3700) as well as the European Union Animals Directive 2010/63/EU and were approved by AU’s Animal Welfare and Ethical Review Body (AWERB).

### Origin of the parasites and their hosts

At the University of Perpignan, we used a Brazilian *Schistosoma mansoni* strain (*Sm*Bre) originally sampled in Recife, Brazil, in the 1960s, provided to our laboratory in 1975 by Pr. Y. Golvan (Faculté de Médecine de Paris—Saint Antoine). It has since then been maintained in its sympatric intermediate host strain *Bg*Bre of the mollusk *Biomphalaria glabrata* (also sampled in Recife in 1975) and *Mus musculus* (SWISS) as definitive vertebrate host. The mollusk strain has albinism from genetic origin, but it does not have any impact on the experimental design. At Aberystwyth University, a Puerto Rican strain (NMRI) of *S*. *mansoni* was used throughout the study and maintained between *M*. *musculus* (Tuck Ordinary; TO) and *B*. *glabrata* (NMRI albino and pigmented hybrid [18]) hosts.

### Maintenance of the life cycle

Miracidia from the *Sm*Bre strain were freshly obtained from eggs extracted from the liver of infected mice. Livers were ground by pestle and mortar and eggs were isolated by sequential filtration with sieves. Isolated eggs were transferred into freshwater and exposed to bright light to stimulate egg hatching. Hatched miracidia were collected by pipetting and frozen in liquid nitrogen after removing as much water as possible.

*Bg*Bre snails were infected using 10 miracidia per snail. Mollusks were screened for infection a month later, and cercariae shed by each positive *B*. *glabrata* were genotyped with sex markers [19, 20]. Seventy cercariae were used to infect a SWISS mouse. All steps of this life cycle were performed as described in [20]. Miracidia from the NMRI strain were also freshly obtained from livers of infected mice (initiated by percutaneous infection of 200 cercariae/mouse; mice were killed at 7 weeks post exposure) and used to maintain the schistosome life cycle (6–8 miracidia per snail) or to initiate drug/miracidia co-culture experiments.

### Biological material

Sporocysts used for chromatin immunoprecipitation followed by sequencing (ChIP-Seq) were obtained through *in vitro* transformation of miracidia. Miracidia were collected for this purpose from freshly hatched eggs extracted from infected hamster livers and placed into wells of a sterile 24-well cell culture cluster (Corning Glass, Corning, NY) containing 15 mL of sterile Chernin's balanced salt solution (CBSS) supplemented with 2X antibiotics (Penicillin-Streptomycin Sigma P4458). Miracidia were cultured at 26°C without light condition for 48h to induce sporocysts transformation. Transformed primary sporocysts (Sp1) were then carefully transferred into a 15-mL sterile tube and centrifuged at 2000 g and 25°C for 10min. Supernatant was discarded and Sp1 were stored at -80°C until further use. Miracidia used for drug co-culture experiments were processed independently, as described below (histone methyltransferase inhibitors study). Data for cercariae and adult worms were obtained from (i) mixed sex infections or (ii) by monomiracidial infection of *Bg*Bre snails [19] and analysis of clonal female cercariae and adults. This latter approach allowed for a more detailed analysis without confounding genotype or sex effects but was done only for the female sex. Cercariae were collected three times from a single snail, 32–54 days after infection by gently pipetting from spring water, avoiding mucus and feces, and subsequently sedimented on ice. Water was removed, and cercariae were kept separately at -80°C before being used for ChIP-Seq. Nine hundred of the same (single sex, clonal) cercariae were used to infect three mice (300 cercariae/mouse), as described in [20]. Mice were sacrificed 42 days later by injection of sodium pentobarbital. Worms were recovered by retrograde perfusions of the hepatic portal system with citrate (7.5%) saline (8.5%) solution administrated through the left ventricle [21]. Worms from each mouse were stored separately at -80°C before being used for ChIP-Seq.

### Histone methylase inhibitors

A Puerto Rican strain (NMRI) of *S*. *mansoni* was used throughout the study and maintained between *M*. *musculus* (HsdOla:MF1) and *B*. *glabrata* (NMRI albino and pigmented hybrid [34] hosts). Cercariae were shed from both *B*. *glabrata* strains by exposure to light in an artificially heated room (26°C) for 1 hour and used to percutaneously infect *M*. *musculus* (200 cercariae/mouse) [22]. At seven weeks post-infection, mice were euthanized and liver eggs were obtained; these eggs were exposed to light to induce miracidia hatching in 1X Lepple water [23]. Following the hatching of miracidia in 1X Lepple water, parasites were collected and enumerated. Subsequent to 15 minutes incubation on ice, miracidia were centrifuged at 700 x g for 5 minutes at 4°C. The miracidia pellet was then resuspended in a small volume of Chernin's balanced salt solution (CBSS) containing 1x penicillin-streptomycin. 500 μL of this miracidia solution, containing ~ 20–50 miracidia, was added to each well of a 48-well culture plate. Epi-drugs targeting *Homo sapiens* G9a/GLP and EZH2 histone methyltransferases (HMTs) were obtained from the Structural Genomics Consortium (http://www.thesgc.org), solubilized in DMSO and subsequently diluted to the correct concentration with CBSS. A 500 μL sample of the relevant drug solution was then added to the treatment wells to give a final concentration of 200 μM (A366, a compound developed for *H*. *sapiens* G9a/GLP HMTs), 20 μM (GSK343, a compound developed for *H*. *sapiens* EZH2 HMT) 10μM (GSK343, A366), 2 μM (GSK343, A366) and 0.4 μM (GSK343, A366) respectively [18, 24–27]. Each treatment was set up in triplicate. Parasites cultured in CBSS with 1% DMSO (final % of DMSO in each treatment well) were set up as controls. Miracidia were then incubated at 26°C in the dark. Since miracidia of the DMSO control had not fully transformed into sporocysts after 24 hours (cilia still attached), the parasites were incubated for a further 24 hours. Following the 48 hours incubation, fully and partially transformed miracidia were enumerated in the DMSO, 2 μM and 0.4 μM cultures. We did not include the 200 μM, 20 μM or 10 μM cultures for the transformation efficiency experiment, as most of the parasites had ruptured and the transformation stage was unidentifiable. An ANOVA followed by *post hoc* analysis with Tukey’s multiple comparison test was performed to infer statistical significance. A miracidium was considered to have fully transformed if all ciliated plates had been shed, and partially transformed if some plates were still attached. Representative images were subsequently acquired at low power (10X objective).

### Chromatin immunoprecipitation and sequencing (ChIP-Seq)

We performed native chromatin immunoprecipitation followed by sequencing (ChIP-Seq) for all developmental stages as described for *S*. *mansoni* in Cosseau et al. [28] (also available online at http://ihpe.univ-perp.fr/methods/methods/native_chip_sm_3.htm).

We used a minimum of 100 miracidia, 100 primary sporocysts, 5,000 cercariae, 2,000 schistosomula and 20 adult worms for each ChIP-seq experiments. For each histone mark, we had three biological replicates for female cercariae and female adults, and two for the other mixed sex developmental stages. Immunoprecipitation was performed using antibodies against H3K4me3 and H3K27me3 on all developmental stages, and H4K20me1 only on female cercariae and female adults. Details for each antibody (supplier, lot number, amount used) are in **Table 1**.

**Table 1:** Details of the antibodies used for ChIP-Seq.

*Antibody*	*Supplier*	*Catalog #*	*Lot #*	*Concentration*	*Amount used*	*Used on*
*H3K4me3*	Diagenode	C15410003	A5051-001P	1.4 μg/μl	4 μL	Miracidia Sp1
	Merck-Millipore	04–745	NG1680351	1 μg/μl	4 μL	Cercariae Schistosomula Adults
*H3K27me3*	Diagenode	C15410069	A1821D/2	1.45 μg/μl	8 μL	Miracidia Sp1 Cercariae Schistosomula Adults
*H4K20me1*	Abcam	Ab9051	GR158874-1	0.7 μg/μl	4 μL	Cercariae Adults

For each sample, we used a control without antibody to assess nonspecific background (bound fraction) and input (unbound fraction). Inputs were used for normalization in all subsequent bioinformatics analyses. Antibodies were carefully tested for specificity as described [27], had been previous shown to be suitable for ChIP-Seq with *S*. *mansoni* [16] and were used in saturation quantities. During the course of the experiments, the anti-H3K4me3 antibody 04–745 of Merck-Millipore was found to be contaminated by the supplier with rabbit DNA and it was not used further. ChIP products from miracidia and Sp1 were sequenced as paired-end 75-bp reads on an Illumina HiSeq 2500 at Wellcome Trust Sanger Institute (UK). The samples were quantified on a high sensitivity bioanalyser, before being cleaned with Agencourt AMPure XP beads. End repair, A-tailing and adapter ligation were performed using the NEB library prep kit, with Agencourt AMPure XP bead cleaning steps between each stage. The amount of template for PCR and the number of PCR cycles required were assessed from a high sensitivity bioanalyser trace post-ligation. Libraries were amplified for 14 cycles. After cleaning with Agencourt AMPure beads, libraries were quantified using a KAPA SYBR FAST ABI Prism qPCR Kit with Illumina GA Primer Premix (10x) and 7 x Illumina GA DNA Standards (Kit code: KK4834) on an ABI StepOnePlus qPCR machine. Libraries were diluted into an equimolar pool and run on a HiSeq 2500, generating 75 base pair, paired end reads.

Cercariae, schistosomula and adult stages were sequenced as single-end 50-bp reads on an Illumina HiSeq 2500 at McGill University and Génome Québec Innovation Centre (http://gqinnovationcenter.com/index.aspx). Briefly, fragmented DNA was quantified using a 2100 Bioanalyzer (Agilent Technologies). Libraries were generated robotically with 10 ng of fragmented DNA (range 100–300 bp) using the Kapa HTP Library Preparation Kit (Kapa Biosystems) as per the manufacturer’s recommendations, except that adapters and PCR primers were diluted 100-fold (final concentration of 0.2 μM). The size selection step was carried out after the PCR step, and the number of PCR cycles was increased by 6. Adapters and PCR primers were purchased from Integrated DNA Technologies and size selection was performed on a Pippin Prep instrument (SAGE Biosciences Inc). Libraries were quantified using the Quant-iT Pico-Green dsDNA Assay Kit (Life Technologies) and the Kapa Illumina GA with Revised Primers-SYBR Fast Universal kit (D-Mark). Average size fragment was determined using a LabChip GX (PerkinElmer) instrument. Cluster formation on the flow cell was performed using the cBot instrument (Illumina) with four indexed libraries per lane. Sequencing, in the form of 50-cycle single-end reads, was performed on a HiSeq 2000/2500 (Illumina) running HCS software version 2.2.38. Demultiplexed FASTQ files were generated by allowing up to one mismatch in the index.

### Quality control, alignment and peak calling

All data processing was performed on a local GALAXY instance (http://bioinfo.univ-perp.fr [29]). Read quality was verified using the FastQC toolbox (https://www.bioinformatics.babraham.ac.uk/projects/fastqc/). All samples had a minimum average read quality score of 30 over 95% of their length, and no further cleaning steps were performed.

Sequences were aligned to the *S*. *mansoni* reference genome v5 [30] with Bowtie v2.1 [31] using parameters–end-to-end,–sensitive,–gbar 4. BAM files generated by Bowtie2 were sorted and then filtered for unique matches with samtools v1.3.1 [32] (samtools view -Sh -q quality value 40–42—F 0x0004 –| grep -v XS:i). PCR duplicates were also removed using samtools (samtools rmdup). Although not mandatory, we found that performing random sampling to use the same amount of uniquely mapped reads for each sample and each histone mark improved sensitivity and specificity when looking for chromatin structure differences. We took 7 million reads for all four histones marks for adults and cercariae, and 1.5–3 million reads for H3K4me3 and H3K27me3 for all other developmental stages. Peak calling using PEAKRANGER v1.16 [33] (parameters: P-value ≤ 0.0001, False discovery rate (FDR) ≤ 0.01, read extension length 200, smoothing bandwidth 99 and Delta 1) was used to visualize histone mark distributions. Input samples were used as a negative control (-c) for normalization. Wiggle files generated in the process were visualized with IGV [34].

### Chromatin landscape

We started our analyses by characterizing the genome-wide chromatin landscape in five developmental stages of *S*. *mansoni*. We used chromstaR (v1.2.0) for peak detection and comparative analysis, using epialleles that chromstaR detected in all replicates of a given developmental stage [35]. ChromstaR is an R package that uses Hidden Markov Models (HMM) to perform computational inference of discrete combinatorial chromatin state dynamics over the whole genome. It can perform uni- or multivariate analyses using several replicates and identifying common and different peaks between conditions [35]. ChromstaR was processed in two steps: (1) we fitted a univariate Hidden Markov Model over each ChIP-seq sample individually (*i*.*e*. each replicate for each developmental stage) and (2) we performed a multivariate HMM over the combined ChIP-seq samples. BAM files from each parasite stage were processed under the combinatorial mode, with a false discovery rate (FDR) cutoff of 0.05 and bin size of 150. We used the input as negative controls for comparative analyses. Transcriptional start sites (TSS) of genes were defined as 3 bp (one base pair upstream and downstream of the +1 transcription site) based on the genome annotation file v.5 downloadable from ftp://ftp.sanger.ac.uk/pub/pathogens/Schistosoma/mansoni/genome/GFF/. A simplified BED file with TSS and transcription end sites (TES) was generated for contigs assembled at chromosome level. Chromosome names were changed to Chr1—ChrW (**S1 File**). Average histone modification profiles were generated over 8,000 bp windows (4,000 bp upstream and downstream of the TSS). Knowing that histone modifications have crucial roles in the regulation of gene expression, we tested the genome-wide coverage (*i*.*e*. the percentage of the genome that is covered by the chromatin state) of each histone mark over the genome and at TSS. In the following, chromatin profiles were generated for fragments over several base pairs (bp) in length. Operationally analytical units of different lengths: ‘bins’ (shortest fragment unit of 150 bp), ‘segments’ (consecutive bins that are in the respective state) and ‘regions’ (consecutive segments separated by short gaps [empty bins with no coverage, generally because these bins are located in repetitive DNA where reads where not uniquely mapped]).

### Identification of stage-specific chromatin differences between female cercariae and adults

We also used chromstaR [35] to find differences present in all three replicates of female cercariae and absent in all replicates of female adults (or *vice versa*). We performed this step for each histone mark separately (univariate) and together (multivariate). The use of a clonal population of cercariae and corresponding adults allowed genotype (and sex)-dependent differences. As each mark has a different distribution, we tested different parameters to assess the best specificity and sensitivity for each histone modification. We noticed after visual inspection that there were two main types of chromatin differences (*i*.*e*. part of the genome when a peak is present/stronger in one developmental stage, and absent/weaker in the other). One type was smaller, spanning 0.3-10kb (segments with no gap) and another type was larger spanning 10–100 kb (regions allowing gaps); we, therefore, defined two sets of parameters to specifically look for these ‘peaks’ (**Table 2**) and 'ranges' (**Table 3**). All differences identified by chromstaR were visually inspected in IGV [34] and then annotated using a combination of the *S*. *mansoni* v5 annotation [30] and an annotation we had produced earlier [17]. We decided to focus our analysis on chromosome scaffolds only (Schisto_mansoni.Chr and Schisto_mansoni.Chr.unplaced), which cover approximately 85% of the genome.

**Table 2:** Parameters used in chromstaR for the detection of ‘peaks’ (300 bp to 10 kb wide). *Bin size*: Size (in bp) in which the genome was fragmented to analyze the histone mark distribution. *Differential score*: value generated by chromstaR which provide an estimation on how divergent two bins are (0 = no difference, 1 = extremely different). *Minimum read count*: minimum number of reads which must be mapped inside a bin in order to take it into consideration. Minimum region length: minimum size (in bp) of consecutive adjacent bins with a different chromatin profile between samples. *False discovery rate* = minimum value to eliminate false positives. *Gap* = size of gaps which are allowed between two bins or group of bins with a different chromatin profile between samples. This is important, as gaps (where no reads are present) are frequent on *S*. *mansoni* genome. The reason for that is only uniquely mapped reads are used, but 47.73% of the genome is repetitive [36].

	*H3K4me3*	*H3K27me3*	*H4K20me1*
*Total uniquely aligned reads*	7,000,000	23,000,000	19,000,000
*Bin size (bin)*	150 bp	150 bp	150 bp
*Differential score (Diffscore)*	0.99	0.99	0.99
*Minimum read count (Diffcount)*	30	30	6
*Minimum region length (minWidth)*	300	300	300

**Table 3:** Parameters used in chromstaR for the detection of ‘ranges’ (10 kb to 100 kb). *Bin size*: Size (in bp) in which the genome was fragmented to analyze the histone mark distribution. *Differential score*: value generated by chromstaR which provide an estimation on how divergent two bins are (0 = no difference, 1 = extremely different). *Minimum read count*: minimum number of reads which must be mapped inside a bin in order to take it into consideration. Minimum region length: minimum size (in bp) of consecutive adjacent bins with a different chromatin profile between samples. *False discovery rate* = minimum value to eliminate false positives. *Gap* = size of gaps which are allowed between two bins or group of bins with a different chromatin profile between samples. This is important, as gaps (where no reads are present) are frequent on *S*. *mansoni* genome. The reason for that is only uniquely mapped reads are used, but 47.73% of the genome is repetitive [36].

	*H3K27me3*	*H4K20me1*
*Total uniquely aligned reads*	7,000,000	7,000,000
*Bin size (bin)*	150 bp	150 bp
*False Discovery Rate (FDR)*	5e^-11^	5e^-6^
*Gap (min.gapwidth)*	25 kb	25 kb
*Minimum region length (width)*	10 kb	10 kb

### Gene ontology enrichment analysis

List of annotated genes with differential chromatin structure between cercariae and adults were tested for statistical overrepresentation in gene ontology (GO) terms using Panther v12 (http://pantherdb.org/geneListAnalysis.do) and GO-slim analyses of molecular functions, biological pathways and protein class under default parameters. We built annotated gene lists depending on the different type of chromatin structure enrichment we saw (*i*.*e*. which mark(s) were enriched at a given position) and the type of differences (peaks, 300 bp to 10 kb, and ranges, 10 kb to 100 kb) in which they were found. When chromstaR detected a gene in both ‘peaks’ and ‘ranges’ (some of these regions were overlapping), we only considered it in the list of ‘ranges’.

### Analysis of histone methylation in repeats

Fastq reads of ChIP-Seq for each stage of the parasite were aligned to the S. mansoni “repeatome” (http://methdb.univ-perp.fr/cgi/download_dump.cgi?file_name=RepBasePerpignanSma52.fasta), a collection of 3,145 consensus repeat sequences [36] using Salmon with default parameters [37]. The ‘Estimated number of reads’ values were used and to avoid division by 0, 1 was added to each count and rounded to the nearest integer. This was used as input to DESeq2 [38] to identify differentially modified repeats. The built-in PCA of DESeq2 was used for graphical representation of the results. Experiments were done in duplicate.

## Results

### Frequency of H3K4me3, H3K27me3, and bivalent methylation in genes shows stage-specific profiles

**Fig 2:**
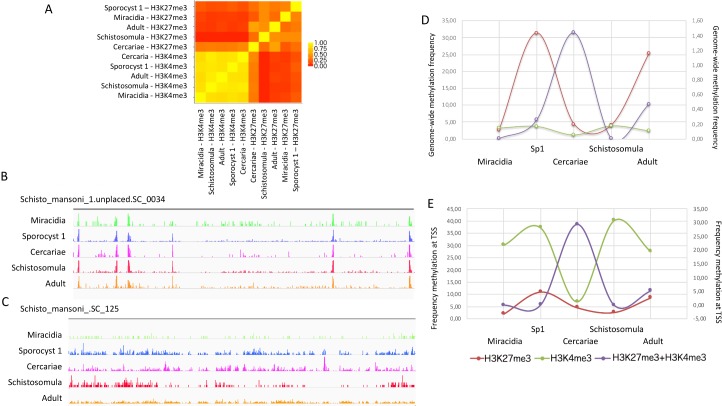
Sample clustering and genome-wide frequency of combinatorial states during the life cycle of *Schistosoma mansoni*. (A) ChIP-seq data heatmap between five parasite developmental stages for H3K4me3 and H3K27me3. Typical example of chromatin profile for H3K4me3 (B) and H3K27me3 (C). (D) Genome-wide frequency of all combinatorial states in all stages. Two waves of repressive H3K27me3 (red line) with a maximum of methylation can be seen in Sp1 and adults, while a constant line of H3K4me3 (green line) is seen throughout the whole cycle (left Y-axis). A single wave of bivalent chromatin (H3K4me3 and H3K27me3 co-localizing, purple line, secondary axis) has its maximum in cercariae (right Y-axis). (E) Frequency methylation at TSS. Intense repressive methylation inside intermediate and definitive hosts (H3K27me3, red line, left Y-axis), intense bivalency state in cercariae (H3K4me3+H3K27me3 at same locus, purple line, secondary axis, right Y-axis) with loss of single active H3K4me3 histone mark (H3K4me3, green line, left Y-axis). X-axis: Five developmental stage of *S*. *mansoni*; Y- axis: Genome-wide methylation frequency (%).

We calculated the genome-wide coverage of trimethylation of histone 3 at lysine 4 (H3K4me3) and at lysine 27 (H3K27me3) in five developmental stages of *S*. *mansoni* (**Fig 2**). First, we analyzed the correlation between all samples to understand how the distribution of both histone marks, spanning the entire genome, changed over all developmental stages. H3K4me3 showed a similar profile between all stages (**Fig 2A and 2B**), with over 60% of peak positions being conserved throughout all stages (**Figs 2A and S1A**). H3K27me3 peaks display a much weaker correlation (0–25% of the peaks conserved between stages) and a very different profile (**Figs 2A and 2C, S1B**), suggesting that this repressive mark is stage-specific. Genome-wide frequency analyses revealed that the presence of both histone marks followed a wave pattern throughout the life cycle. Simultaneous presence of two histone modifications (H3K4me3 and H3K27me3), occur in 5.8%-35.2% of *S*. *mansoni* genome from miracidia to adult worms. Genome-wide, H3K27me3 accumulates in Sp1 and adult stages, and is relatively low during miracidia, cercariae and schistosomula stages (**Fig 2D**). When not associated with another mark that we have studied, H3K4me3 is stable throughout the life cycle and only slightly increases during the Sp1 and schistosomula stages (**Fig 2D**). Bivalent marks (presence of both H3K4me3 and H3K27me3 at the same position, possibly on the same nucleosome) occur in Sp1, cercariae and adults, but were at the highest level at the cercarial stage. The pattern of active H3K4me3 and repressive H3K27me3 is different over the whole genome and showed some specificity when focused at the TSS (**Fig 2E**). Throughout the schistosome life cycle, two sharp increases in H3K4me3 at the TSS can be seen (peaking during Sp1 and schistosomula). Similarly, H3K27me3 at the TSS also displays two peaks found in both Sp1 and adult stages. Bivalent marks at the TSS are at their maximum during the cercarial stage (**Fig 2E**).

**Fig 3:**
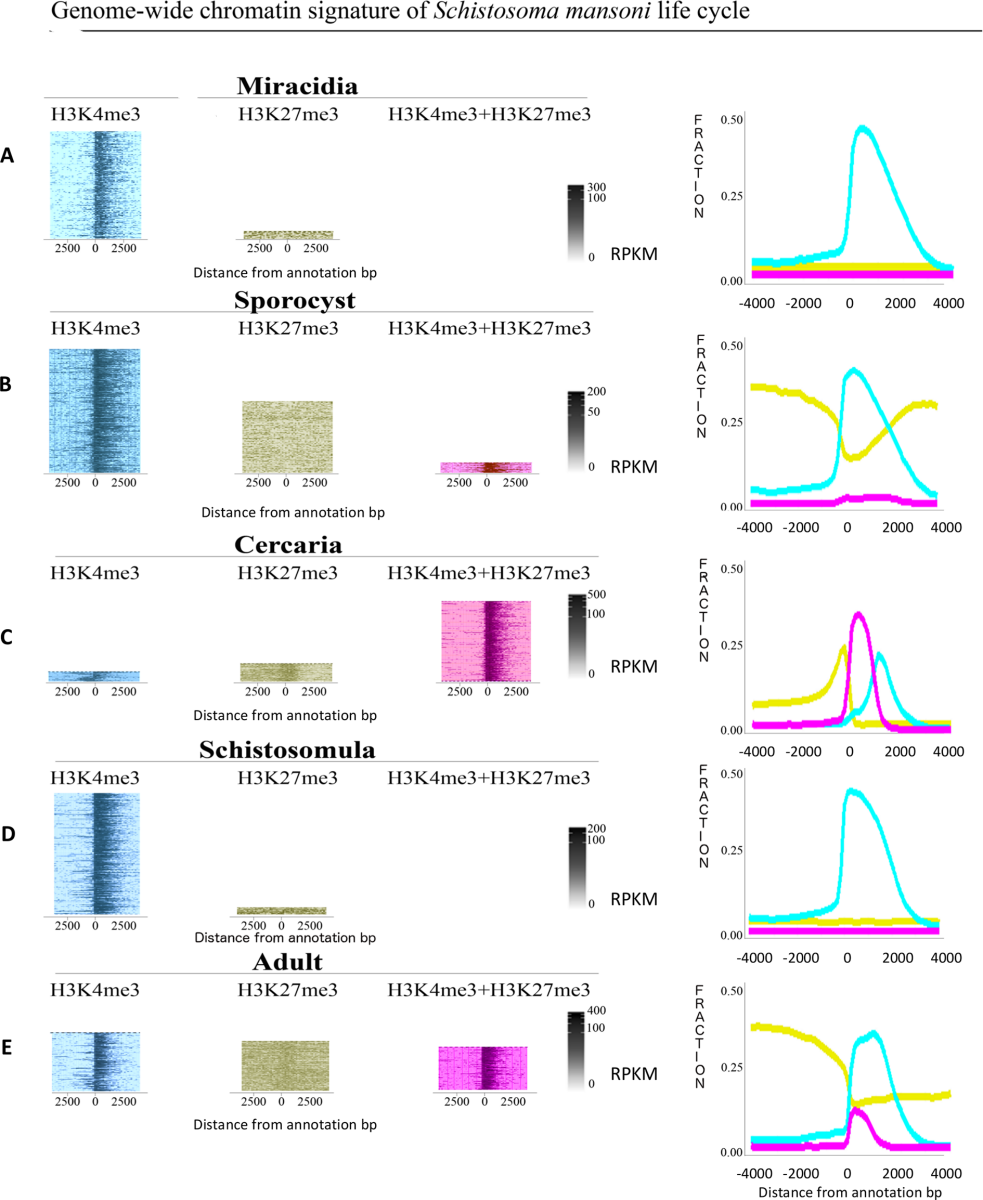
Heatmap and enrichment of chromatin states around transcription start sites (TSS) of five developmental stages of the *Schistosoma mansoni* life cycle. (A) Heatmap of miracidia stage for H3K4me3, H3K27me3 and H3K4me3+H3K27me3. (B) Heatmap of sporocyst stage for H3K4me3, H3K27me3 and H3K4me3+H3K27me3. (C) Heatmap of cercaria stage for H3K4me3, H3K27me3 and H3K4me3+H3K27me3. (D) Heatmap of miracidia stage for H3K4me3, H3K27me3 and H3K4me3+H3K27me3. (E) Heatmap of miracidia stage for H3K4me3, H3K27me3 and H3K4me3+H3K27me3. Logarithms (observed/ expected) for each histone modification were plotted along *S*. *mansoni* genome and 4 kb upstream and downstream for each stage (A-E). Colors: Blue = H3k4me3; Yellow = H3K27me3; Purple = H3K4me3+H3K27me3.

Next, we looked at how histone marks were distributed around the transcription start sites (TSS) of genes (**Fig 3**). In free-swimming miracidia, we detected the presence of H3K4me3 over 36.8% of TSS and H3K27me3 over 2.2% of TSS. H3K4me3 segments cover 11,834 kb of the genome and starts around the TSS and do not spread more than 3,000 bp downstream of the TSS (**Fig 3A and Table 3**). When the miracidium loses its ciliated surface to become a Sp1, H3K4me3 increases to be present at 38.7% of TSS. H3K27me3 also increases to be present over 15.5% of TSS (covering 155,278 kb, mainly 2,000 bp upstream and downstream of TSS). Also, we observed that bivalent methylation starts in Sp1 (although at a very low level) spanning -2000 bp to +500 bp in 1.78% of the TSS (**Figs 2D and 3D**).

The transition to cercariae is characterized by a notable increase in bivalent marks (H3K4me3 and H3K27me3), occurring at 32.7% of all TSS (-500 to +1000 bp). In cercariae, H3K4me3 covers 9,990 kb of the genome (5.22% of TSS), starting around 500 bp upstream of TSS. For the broad H3K27me3, 32,756 kb of the genome are covered (5.54% of TSS), slightly upstream of the gene body, and decreases while the bivalency increases around TSS (**Fig 3C**). After the cercaria to schistosomula transformation, H3K4me3 reaches coverage levels of 41.81% at TSS. Also, a strong loss of H3K27me3 is observed with only 2.93% of TSS covered, and no bivalent state observed around TSS (**Fig 3D**).

In adults, 60 days post-infection, a notable increase in trimethylation of H3K27 is observed (**Fig 3E**). H3K27me3 covers 133,653 kb and the coverage at TSS increases 4-fold inside the definitive host in comparison to the previous stage (from 2.93% in schistosomula to 14.38% in adults). The active H3K4me3 mark covers 10,493 kb (24.1% of TSS) and starts around 500 bp upstream while the repressive HK27me3 mark decreases. The bivalent methylation in adult worm (-500 to +1000 bp) reaches 10% of coverage at TSS (**Table 4**).

**Table 4:** Description of frequency and covered chromatin state (kb) in miracidia, Sp1, cercariae, schistosomula and adults for H3K4me3, H3K27me3 or both (bivalent state), genome-wide and at TSS (Transcription Start Site of genes).

	*Miracidia*	*Sp1*	*Cercariae*	*Schistosomula*	*Adults*
*Covered histone marks over whole genome*	**Genome-wide**	**Genome-wide**	**Genome-wide**	**Genome-wide**	**Genome-wide**
***H3K27me3***	*10*,*685 kb*	*155*,*278 kb*	*32*,*756 kb*	*16*,*159 kb*	*113*,*653 kb*
***H3K4me3***	*11*,*834 kb*	*14*,*423 kb*	*9*,*990 kb*	*13*,*938 kb*	*10*,*493 kb*
*Frequency of chromatin state over whole genome*	**Genome-wide**	**Genome-wide**	**Genome-wide**	**Genome-wide**	**Genome-wide**
***H3K27me3 only***	*2*.*70%*	*31*.*36%*	*4*.*32%*	*4*.*13%*	*25*.*34%*
***H3K4me3 only***	*3*.*16%*	*3*.*62%*	*1*.*18%*	*3*.*76%*	*2*.*35%*
***H3K27me3 & H3K4me3***	*0*.*00%*	*0*.*26%*	*1*.*44%*	*0*.*01%*	*0*.*47%*
***Total methylated sites***	*5*.*86%*	*35*.*24%*	*6*.*94%*	*7*.*90%*	*28*.*16%*
*Frequency of chromatin state at TSS*	**At TSS**	**At TSS**	**At TSS**	**At TSS**	**At TSS**
***H3K27me3 only***	*2*.*29%*	*15*.*59%*	*5*.*54%*	*2*.*93%*	*14*.*38%*
***H3K4me3 only***	*36*.*87%*	*38*.*71%*	*5*.*22%*	*41*.*81%*	*24*.*17%*
***H3K27me3 & H3K4me3***	*0*.*00%*	*1*.*78%*	*32*.*79%*	*0*.*0%*	*10*.*04%*
***Total methylated sites***	*39*.*16%*	*56*.*08%*	*43*.*55%*	*44*.*74%*	*48*.*59%*

### Pharmacological inhibition of H3K27 methyltransferases arrests miracidium to sporocyst transition

**Fig 4:**
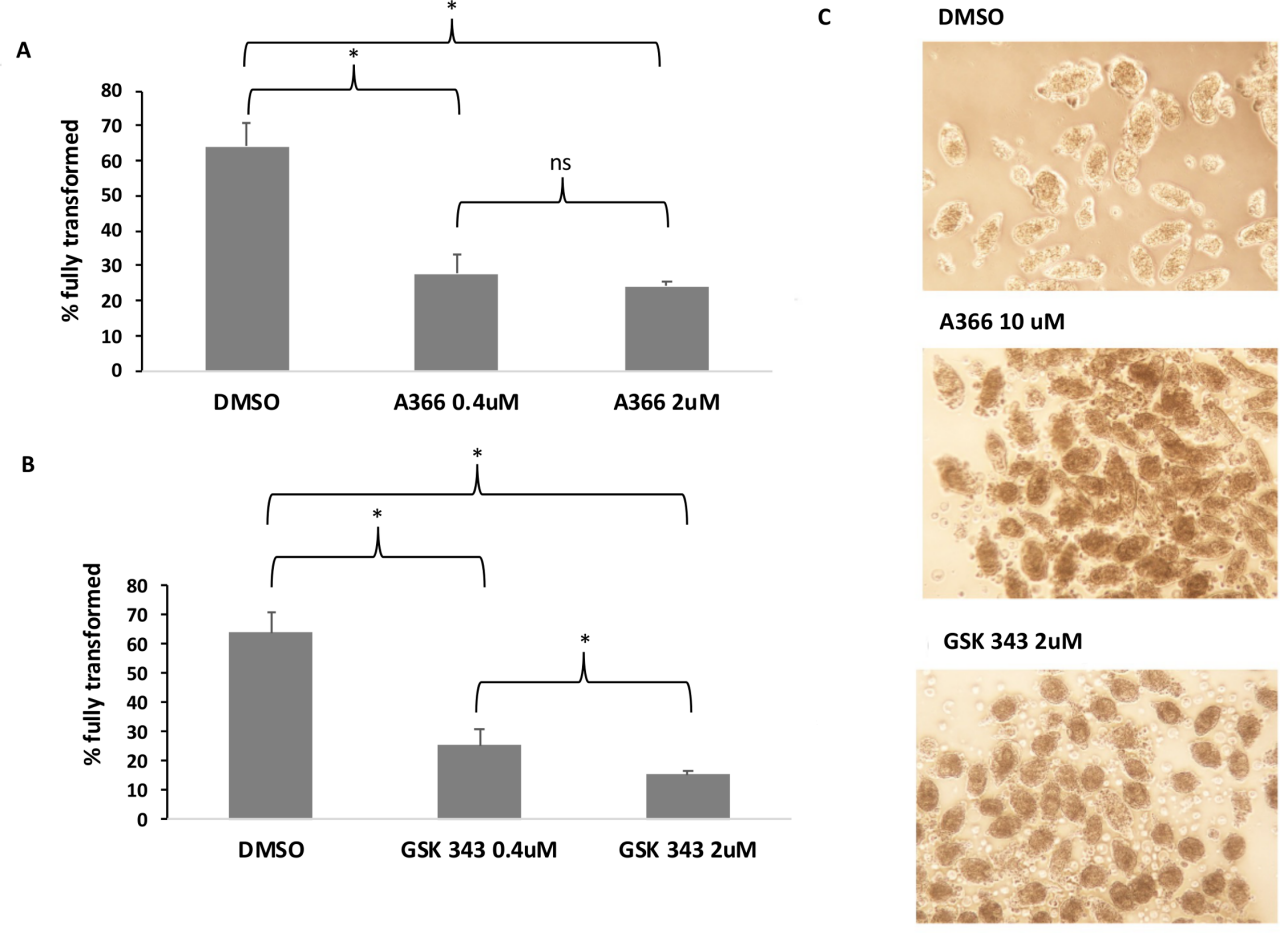
Histone methyltransferase inhibitors block miracidium to primary sprorocyst transformation. Each treatment was set up in triplicate and parasites were cultured in CBSS with 1% DMSO at a controlled temperature of 26°C (in the dark). An ANOVA followed by *post hoc* analysis with Tukey’s multiple comparison test was performed to infer statistical significance; *p<0.005. **A**. Effect of 0.4 μM of A366, 2 μM of A366 and DMSO (negative control). **B**. Effect of 0.4 μM of GSK343, 0.4 μM of GSK343 and DMSO (negative control). **C.** Photomicrographs of miracidia to sporocyst transition in the presence of histone methyltransferase inhibitors. Miracidia transformed in the presence of DMSO (negative control) show normal transformation into sporocysts, but fail to lose their ciliated plates and do not develop into primary sporocysts in the presence of of A366 and GSK343 (10 μM). Representative images were acquired at low power (10X objective).

Since our chromatin landscape studies had identified trimethylation of H3K27 as a histone modification that undergoes major modifications during the life cycle, we wondered if histone modification would be cause or consequence of stage to stage progression. We reasoned that if pharmacological inhibition of this modification results in blocking the life cycle then histone modifications are cause of the developmental plasticity. We decided to test this hypothesis at the miracidium to sporocyst transition where the increase in H3K27 trimethylation is most pronounced (**Fig 2D**) and the environment can be easily controlled. Specifically, we used drugs targeting *H*. *sapiens* G9a/GLP (A366) and EZH2 (GSK343) H3K27 methyltransferases to assess the ability of these epidrugs to block transformation efficiency (**Fig 4**). In the presence of both drugs, the miracidium to sporocyst transformation efficiency was significantly reduced when compared to the DMSO controls, even at concentrations as low as 0.4 μM. In DMSO controls, 64% of miracidia fully transformed into sporocysts while exposure to 0.4 μM and 2 μM of A366 significantly reduced transformation efficiency to 28% and 24%, respectively (p<0.005). Additionally, for GSK343, there was a dose-dependent decrease in transformation efficiency, as there was a significant difference (p<0.005) in transformation efficiencies between the 0.4 μM (21% fully transformed) and 2 μM (17% fully transformed) cultures (**Fig 4**). These results suggest that changes in H3K27me3 (and probably histone modification changes in general) are essential for life cycle progression in *S*. *mansoni*.

### Differences between female cercariae and adults are concentrated around TSS of genes

To gain further insights into chromatin modifications in the transition that is of principal medical interest, *i*.*e*. female cercariae to female adults, we also performed ChIP-seq on H4K20me1, a mark associated with the transcriptional activation state and that could be involved in bivalency with repressive H3K27me3 in early vertebrate embryos [39]. Knowing that each histone mark has a specific profile, two set of parameters were used to detect small peaks (< 10 kb), as well as differences over ranges (10–100 kb) between cercariae and adults (detailed in **Tables 2 and 3)**. We considered only strong differences (which we also call “differential enrichment”), characterized by the presence/absence in one stage and its absence in another, or when the enrichment in one condition is significantly more intense in one stage (differential score > 0.99 in chromstaR). To target the gender that has the highest impact on host morbidity (driven by parasite egg production), we focused our generation of ChIP-Seq data on female and maximized the signal for this analysis (*i*.*e*. to detect potentially important female specific epialleles).

Using chromstaR, we investigated 6,182 peaks (covering 17,683 kb) for H3K4me3, 22,562 (covering 163,424 kb) for H3K27me3 and 20,468 (covering 172,714 kb) for H4K20me1, to spot significant differences between female cercariae and female adults. We identified a total of 1,195 peaks (< 10 kb) where at least one histone mark showed a differential enrichment between cercariae and adults (see **S2 File** for a detailed identification of all these regions). In 14% of the cases (169), two or more marks displayed a different profile (at a given position, one could be enriched in adults and the other in cercariae, or both could be enriched in a given developmental stage). Details about the distribution of the differential enrichment are in **Table 5**. The most striking feature is that the vast majority of the differences for all three marks occur with a peak or stronger peak in cercariae. 82% of the differences involving H3K4me3, 96% of the differences in H3K27me3 and 92% of the differences in H4K20 are characterized by new peaks or peaks that are stronger in cercariae. These differentially enriched regions are preferentially located within annotated genes or transcripts [34]. Differential enrichments in H3K4me3 were located around transcription start sites (TSS) of genes 68% of the time in female cercariae and 61% of the time in female adults.

**Table 5:** Description of the distributions of peaks (0.3–10 kb) between cercariae and adults for each histone mark. TSS = Transcription Start Site of genes. Multiple = H3K4me3 + H3K27me3 + H4K40me1 at the same locus. * In the case of H4K20me1, most of the marks were wide and often covering a large part of the gene, if not the whole gene.

*Histone marks*	*Peaks/stronger peaks in cercariae*			*Peaks/stronger peaks in adults*		
	Total	*In genes*	*At TSS*	Total	*In genes*	*At TSS*
*Total H3K4me3*	455	*425 (93%)*	*310 (68%)*	97	*79 (81%)*	*57 (59%)*
*Total H3K27me3*	122	*80 (71%)*	*17 (15%)*	5	*4 (80%)*	*2 (40%)*
*Total H4K20me1*	323	*317 (98%)*	309* (96%)	43	*42 (95%)*	*16 (36%)*
*H3K4me3 & H3K27me3*	16	*13 (81%)*	*8 (50%)*	10	*8 (80%)*	*6 (60%)*
*H3K27me3 & H4K20me1*	22	*16 (72%)*	*7 (31%)*	7	*6 (85%)*	*4 (57%)*
*Multiple*	8	*6 (75%)*	*5 (62%)*	3	*3 (100%)*	*1 (33%)*

### Ranges with a unique chromatin structure in female cercariae are enriched in development related genes

While performing visual inspection to validate the differentially enriched regions identified by chromstaR, we observed wide ranges (from 10 to 100 kb long) with a differential chromatin structure (see **S2 Fig** for an example). We adjusted chromstaR parameters to specifically detect these wide regions. We found 851 of them (from 10 to 100 kb long) in cercariae, and only 15 of them in adults. In cercariae, 488 were enriched in H4K20me1, 186 in H3K27me3 and 177 in both. These ranges contained at least one gene, with the exception of regions enriched in both H3K27me3 and H4K20me1 (**Table 6**). Genomic coordinates of these regions are available in **S3 File** and gene lists present in these regions can be consulted in **S4 File**.

**Table 6:** Description of the large differentially enriched regions (ranges, 10–100 kb) between cercariae and adults for each histone mark combination, as well as the number of annotated genes present in these regions and the number of regions without any coding genes (and their cumulated length). tRNAs genes were excluded from the gene counts. We also calculated the total length of these regions for each mark.

*Histone marks*	*Enrichment in cercariae*				*Enrichment in adults*			
	# of regions	# of genes	#of regions without genes	Tot. length	# of regions	# of genes	#of regions without genes	Tot. length
*H3K27me3 only*	186	242	37 (808 kb)	4,796 kb	9	8	3 (53 kb)	205 kb
*H4K20me1 only*	500	656	69 (1,450 kb)	17,974 kb	5	4	1 (27,45 kb)	91 kb
*H3K27me3 & H4K20me1*	177	147	56 (936 kb)	10,993 kb	1	0	1(27 kb)	27 kb

We were curious to know if genes present in differentially enriched chromatin regions would present specific molecular or biological functions. To investigate this matter, we performed gene ontology overrepresentation tests. To make our analysis as precise as possible, we tested different gene lists depending on the chromatin structure present at the differentially enriched regions (**Table 7**). We separated genes found in peaks from the ones found in ranges. When chromstaR found the same gene in both peak and range region, we excluded it from the peak. There was no overrepresentation in adults at all. This was not surprising due to the relatively low number of enriched regions in adults, when compared to cercariae. In peaks identified in female cercariae, we only found overrepresentation of GO terms associated with regulation of transcription to contain the repressive H3K27me3 mark. In ranges, we found that the presence of H4K20me1 is strikingly associated with developmental functions such as homeobox transcription factors, regulation of transcription, and the NOTCH pathway.

**Table 7:** Gene ontology overrepresentation depending on the type of chromatin enrichment. In short regions, we removed genes that had also been identified in the long region analysis. Symbol code: *Panther Pathways, ** = Biological Process, *** = Molecular Function, **** = Protein Class. Short regions: 300 bp– 10 kb. Long regions: 10 kb– 100 kb.

	*Enriched marks*	*Number of Genes*	*GO terms overrepresentation?*
***Cercariae in peaks***	*H3K4me3*	214	No
	*H3K27me3*	30	**Regulation of transcription from RNA polymerase II promoter (GO:0006357) ***Seq.-specific DNA binding transcription factor activity (GO:0003700), DNA binding (GO:0003677), Nucleic acid binding (GO:0003676), Binding (GO:0005488) ****Zinc finger transcription factor (PC00244), DNA binding protein (PC00009), Transcription factor (PC00218)
	*H4K20me1*	66	No
***Cercariae in ranges***	*H3K27me3 only*	231	No
	*H3K27me3* *H3K4me3 peaks at TSS*, *but are not enriched compared to adults*	3	No
	*H3K27me3* *H3K4me3 peaks at TSS*, *and are enriched compared to adults*	8	No
	*H4K20me1 only*	472	*Notch signalling pathway (P00045) **Nervous system development (GO:0007399), Regulation of transcription from RNA polymerase II promoter (GO:0006357), Developmental process (GO:0032502), Multicellular organismal process (GO:0032501), Single-multicellular organism process (GO:0044707), Regulation of nucleobase-containing compound (GO:0019219), Organelle organization (GO:0006996) ***Seq.-specific DNA binding RNA polymerase II transcription factor (GO:0000981), Seq.-specific DNA binding transcription factor activity (GO:0003700), DNA binding (GO:0003677) ****Homeobox transcription factor (PC00119), Helix-turn-helix transcription factor (PC00116), Transcription factor (PC00218)
	*H4K20me1* *H3K4me3 peaks at TSS*, *but are not enriched compared to adults*	40	No
	*H4K20me1* *H3K4me3 peaks at TSS*, *and are enriched compared to adults*	144	**Anatomical structure morphogenesis (GO:0009653)
	*H3K27me3 and H4K20me1*	128	**Segment specification (GO:0007379), Pattern specification process (GO:0007389), Cell differentiation (GO:0030154), Regulation of transcription from RNA polymerase II promoter (GO:0006357), Developmental process (GO:0032502), Regulation of nucleobase-containing compound process (GO:0019219) ***Seq.-specific DNA binding RNA polymerase II transcription factor (GO:0000981), Seq.-specific DNA binding transcription factor activity (GO:0003700), DNA binding (GO:0003677), Nucleic acid binding (GO:0003676), Binding (GO:0005488) ****Homeobox transcription factor (PC00119), Helix-turn-helix transcription factor (PC00116), Transcription factor (PC00218), DNA binding protein (PC00009), Nucleic acid binding (PC00171)
	*H3K27me3 and H4K20me1* *H3K4me3 peaks at TSS*, *and are enriched compared to adults*	8	No
	*H3K27me3 and H4K20me1* *H3K4me3 peaks at TSS*, *and are enriched compared to adults*	10	****Cytokine receptor (PC00084)

### Chromatin landscape of repetitive DNA elements is stage-dependent

**Fig 5:**
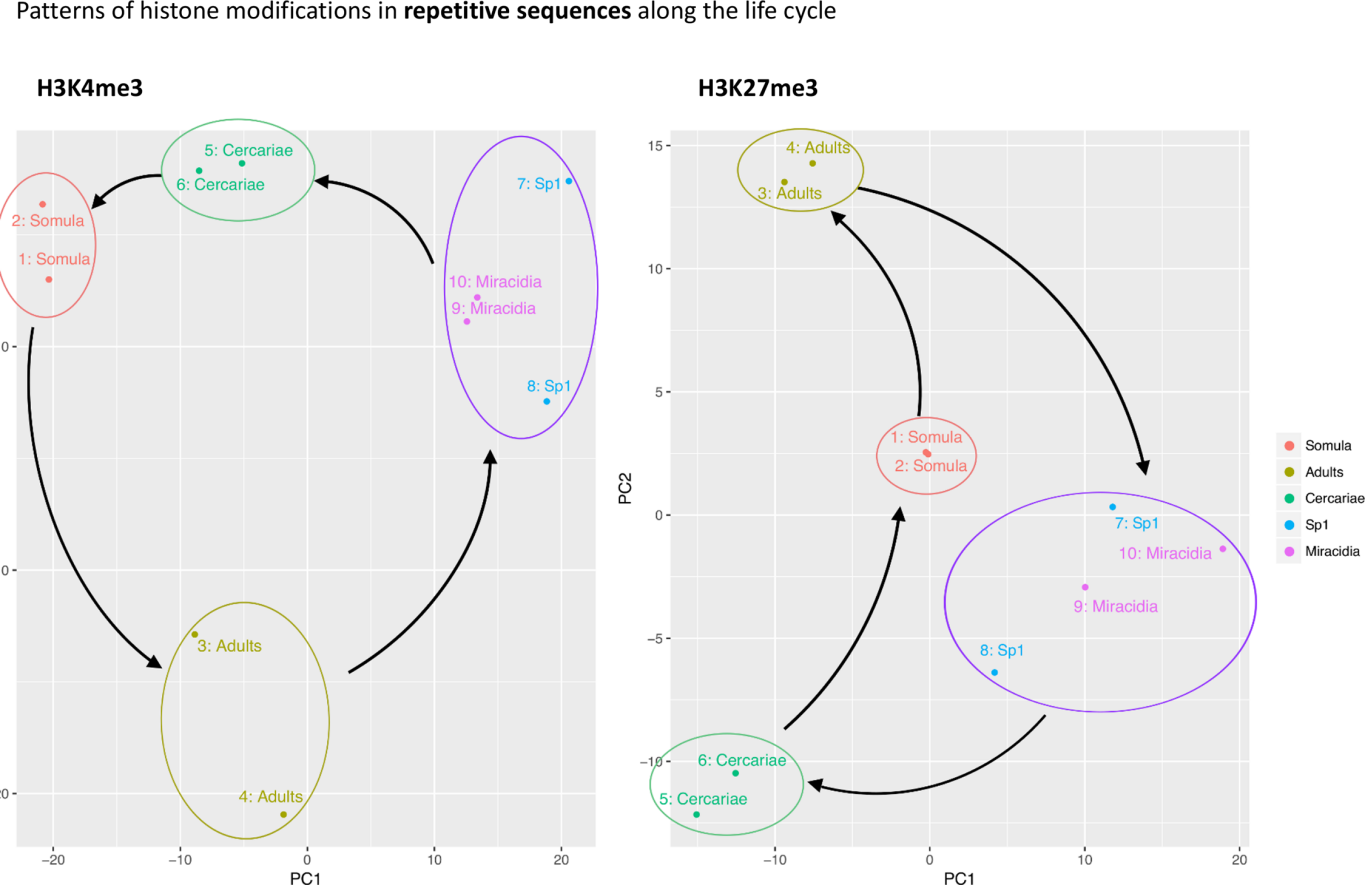
Principal component analysis (PCA) of ChIP enrichment for H3K4me3 (left) and H3K27me3 (right) over repetitive sequences. All life cycle stages segregate in the PCA but miracida and Sp1 (separated by 48 hours) are very close and could only be differentiated by H3K4me3 methylation. Arrows indicate developmental direction and are there to guide the eye.

The repeatome of *S*. *mansoni* shows clear stage dependent histone methylation differences (**Fig 5**). Stages can be segregated by histone methylation on repeats alone. However, for miracidia and Sp1, in contrast to histone modifications in the TSS, histone methylation at repeats is very similar. This indicates that modification of histone methylation in TSS in miracidia to Sp1 is very specific.

## Discussion

Here, we describe for the first time the dynamic nature of two histone modifications throughout the five major developmental stages of a multicellular human pathogen. There are clear distinctions in the histone modification pattern between the stages, and interestingly, we observe that both studied marks follow a cyclic distribution throughout the parasite’s life cycle.

In the literature, H3K4me3 is known to be associated with promoters and transcription start sites of transcriptionally competent genes of vertebrates and invertebrates [40–44]. This mark is generally considered a transcriptional activator, despite some recent evidence hinting that it could be a by-product of transcription instead [45]. We had previously shown in *S*. *mansoni* cercariae and adults that this mark was also preferentially distributed around TSS of genes, similarly to what is found in other metazoans [16]. We can now confirm that this particular histone modification is found at the TSS in all other analyzed schistosome life cycle stages. Interestingly, the presence of H3K4me3 alone (without H3K27me3) is relatively stable when the entire genome is considered (**Fig 2D**), but when we focus on TSS only, we see a strong decrease of solely H3K4me3 in cercariae (**Fig 2E**). In this stage, most TSS have bivalent methylation and transcription is probably poised there. This fits the low transcription level in this developmental stage [16].

H3K27me3 is generally described as a mark associated with facultative heterochromatin (*i*.*e*. genomic regions that become heterochromatic in certain cells, tissues, or depending on the developmental processes), which impedes transcription [46–48]. In *S*. *mansoni*, the genome-wide distribution of this mark broadly fluctuates throughout development, with two peaks occurring at the Sp1 and the adult stages. In contrast, this mark is globally very low in miracidia and schistosomula. In cercariae, the mark co-localizes with H3K4me3 (**Fig 2D and 2E**). In zebrafish, fruit flies and mice, the removal of H3K27me3 plays an important role in key developmental stages, tissue regeneration and cell differentiation [49–51]. We can, here, hypothesize a similar function in *S*. *mansoni* for transition from cercariae to schistosomula. In the case of primary sporocysts, in which cells multiply at a high rate to produce hundreds of secondary sporocysts and then mature in cercariae, the addition of H3K27me3 to H3K4me3 at TSS probably promotes cell growth without differentiation [52]. Several genes identified in proliferating sporocyst cells share molecular signatures with neoblast-stem cells genes, mainly planarians neoblasts [53]. Wang et al. [53] had found induction of transcription for a small group of genes when miracidia developed into Sp1. These genes include *Vasa-like* (Smp_068440, Smp_154320, Smp_033710) that are required for proliferation and expansion of neoblasts, putative polo kinase (Smp_009600) that is probably activating mitosis, and fibroblast growth factor receptor-encoding genes (Smp_157300, Smp_175590) that are potentially required for cell cycle and DNA repair machinery [53]. The gene expression observed for these genes during miracidium to sporocyst transition increased from 1.6 to 4.8-fold. Interestingly, we detected an increase of H3K27me3 presence around their TSS. Basically, we see a very simple chromatin structure in and around TSS in miracidia (presence or absence of H3K4me3) but the situation becomes more complex in the Sp1 stage. Specific chromatin remodeling seems to be necessary for initiation of transcription.

The presence of the *a priori* antagonistic H3K4me3 and H3K27me3 at the TSS of genes has been described several times in embryonic stem cells and is considered as a mechanism to poise transcription at specific loci, being silenced and ready to engage either activation or inactivation upon removal of the respective mark [51–52,54]. We had already proposed the presence of these bivalent marks, associated with an absence of transcription, in cercariae [16]. Throughout the life cycle of the parasite, this bivalency is predominant in the very short-lived cercarial stage. As both miracidia and cercariae have a similar life expectancy (16–24 hours at 25°C after being released in water) [55–56], we initially hypothesized that both life stages would have a similar chromatin structure regarding these bivalent modifications; however, this is clearly not the case. It could be that this observation is linked to how miracidia and cercariae are generated during schistosome development. For example, miracidia are the result of sexual reproduction and a significant demethylation of repressive H3K27me3 is observed during this process. A similar mechanism is observed in mammalian reproduction with intense reduction of H3K27me3 during the formation of the zygote followed by an increase of methylation of H3K27me3 at blastocyst stage [57]. With this in mind, an interesting perspective would be to explore the chromatin landscape of “intermediate” developmental stages, such as unhatched miracidia, to get a more precise picture and understanding of histone changes at the very early stages of the flatworm development. In *S*. *mansoni*, we also noted a particularly marked methylation of H3K27 in accordance to cell development during the transition from miracidia to primary sporocyst (Sp1). On the other hand, cercariae are a product of asexual multiplication within secondary sporocysts (Sp2). It is possible that, in *S*. *mansoni*, bivalent histone marks are only involved in asexual multiplication. It would be interesting to see if this bivalency in cercariae is conserved in other *Schistosoma* species. Miracidia actually have the lowest amount of bivalent marks (and H3K27me3 in general) throughout the whole lifecycle suggesting a more euchromatic situation in general. We have previously demonstrated that histone deacetylation inhibitors can reversibly inhibit miracidia to sporocyst transition, suggesting that heterochromatisation is important during this step [58]. Here, we additionally demonstrate that pharmacological inhibition of histone methylation by A366 and GSK343 also blocks sporocyst development. It had been previously shown that inhibition of EZH2, via GSK343, also blocks schistosomula development [59,60]. In both studies, EZH2 histone methyltransferase activity (H3K27me3) appears essential for lifecycle progression. While A366 also blocks miracidia transformation, there is no clear G9a/GLP ortholog in the current *S*. *mansoni* genome assembly. This would suggest that A366 exerts its activity via promiscuous inhibition of other schistosome HMTs (responsible for H3K27me3 or other modifications) [61, 62]. Nevertheless, our results suggest that HMT activity is essential for parasite development and this class of enzyme represents a suitable drug target.

We wanted to better characterize the chromatin structural changes between cercariae and adults, as this transition is fundamental in the epidemiology of the parasite, and a better understanding could lead to the discovery of new therapeutic targets. Our laboratory has previously demonstrated that in male cercariae H3K27 trimethylation around TSS is much stronger than in female cercariae and that there are pronounced changes in H3K27me3 associated with transition into adult males [17]. For this investigation, we chose to work on female worms only, also to have a clearer signal (*i*.*e*. without confounding potentially sex-specific effects) and because females play a crucial role in morbidity of the mammal host, through egg production. A possible caveat of our experiment is that we obtained female adults from single sex, clonal infection. Hence, the females we collected are not fully mature, and the chromatin structure we observe will not show any specific chromatin changes corresponding to sexual maturation. Also, for both cercariae and adults, we used a very limited number of genotypes due to clonal infections. In total, we had two female genotypes (one was done in replicate). The objective was to have a better, clearer signal and to see how the chromatin structure from a single genotype would evolve from one developmental stage to another. As the *Schistosoma Sm*Bre strain that we used has a very low genetic diversity, and we previously observed very low epigenetic difference between these two genotypes [16], we believe that the results can be transposed to the whole strain. We also added a third epigenetic mark, H4K20me1. This histone modification has several distinct functions in the genome: ensuring the genome integrity and maintenance (DNA repair, replication and compaction [63]), quiescence [64], is associated with high transcriptional levels [65] and plays essential roles in development in drosophila and mouse [43,66]. As we were working with whole organisms composed of variety of tissues and cell types, we chose to focus on very strong differences (very often characterized by the presence of a peak in one stage and the absence in another, or at least two-fold differences in peak height), meaning that they would be present in most of the tissues. We looked for two types of differences: peaks (300 bp to 10 kb, usually a single peak) and ranges (10 to 100 kb, corresponding to groups of peaks). The rationale behind this was that typical parameters from chromstaR to identify differentially enriched regions were not efficient on the *S*. *mansoni* genome due to its high repetitive DNA content (where no sequence reads are mapped) and, therefore, peak calling parameters needed to be adjusted. The number of differential peaks is relatively high in H3K4me3 (552 out of 6,192 peaks, 8.91%) but much lower for H3K27me3 (85 out of 22,562 peaks, 0.38%) and H4K20me1 (366 out of 20,468 peaks, 1.79%). These differences are mainly characterized by the presence of a peak or a stronger peak in cercariae and its absence or reduced size in adults (82.4%, 94.1% and 88.2% for H3K4me3, H3K27me3 and H4K20me1, respectively). These changes occur mostly around the transcription start sites of genes, hinting at a possible effect on gene regulation. No specific pathway was found, but there is an over-representation of genes involved in regulation of transcription and DNA binding in regions with increased H3K27me3 in cercariae. The presence of this repressive mark in genes involved in transcription control fits our previous observation regarding the poised transcriptional state in cercariae. We then looked at the ranges (10 to 100 kb long), which were differentially enriched in H3K27me3, H4K20me1, or both. Once again, most of the enrichments occur in cercariae (meaning that peaks are much more intense in this stage, when compared to adults). Ranges with differential enrichment in cercariae of H4K20me1 alone or combined with H3K27me3 present an overrepresentation of genes involved in regulation of transcription and developmental processes. We also highlighted the presence of the Notch signaling pathway in regions differentially enriched in H4K20me1 in cercariae. Notch signaling is a very well conserved metabolic path in metazoans, involved in embryonic development, cell lineage and tissue regeneration [67]. Interestingly, mRNA presence of homeotic genes was found to be different between cercariae and adults already in the comparative transcriptomics part of Picard et al. [17] and also the Notch signaling pathway had emerged as potentially important factor for sex specific differences there. Our results lend further support to the idea that chromatin structure differences in developmental genes could be the origin of such sex-specific differences in developmental trajectories and the matter shall be analyzed in more detail in the future.

There are not many differences between the overrepresented gene ontology terms in ranges of H4K20me1 only or associated with H3K27me3 (**Table 7**), but we can assume that different mechanisms could be associated with these differential chromatin structures. For example, genes marked with one of the two chromatin mark combinations could be activated faster than the other upon penetration of the cercariae in the host. We found little information regarding the co-localization of H4K20me1 and H3K27me3 over such ranges, only that in *Drosophila* it was involved in early embryo development [38,42]. This should be investigated further in *Schistosoma*. The presence of ranges with differential enrichment was previously described with H3K27me3 in mouse embryonic fibroblast and associated with gene-poor and transposable element rich regions [68]. However, to our knowledge, no such observation was described for H4K20me1 until now. For operational reasons, and to avoid confounding genotype dependent effects, we used a clonal population for the detailed analysis of cercaria to adult transition. Further studies will show if our results apply also to the males or are specific to females. Consequently, histone modifying enzymes that change the methylation status of H3K27me3 and H4K20me1, and potentially prevent the development of female cercariae into female adult worms, emerge as drug targets from this study. Our study shows that all the five developmental stages studied here present a specific chromatin structure. It indicates that deposal of the permissive H3K4me3 and repressive H3K27me3 are carefully controlled along the genome. This makes the enzyme complexes that generate ("writers") or read ("readers") these modifications suitable targets to control development of the parasite.

### Data availability statement

Chromatin landscapes of the different life cycle stages are available at the *Schistosoma mansoni* genome browser of http://genome.univ-perp.fr and as TrackHub at http://ihpe.univ-perp.fr/acces-aux-donnees/.

ChIP-Seq reads are available at the NCBI-SRA under the BioProjects numbers PRJNA219440 and PRJNA236156. Details are available in **Tables 8 and 9**.

**Table 8:** Details for the mixed sexes ChIP-Seq reads available at the NCBI-SRA.

*Library*	*Biosample*	*Strain*	*Sex*	*Antibody*	*Replicate 1*	*Replicate 2*
Miracidia	SAMN06837687	SmBRE	Mixed	H3K4me3	SRR6307186	SRR6307187
	SAMN06837688	SmBRE	Mixed	H3K27me3	SRR6307190	SRR6307191
Sp1	SAMN08039006	SmBRE	Mixed	H3K4me3	SRR6307188	SRR6307189
	SAMN08039006	SmBRE	Mixed	H3K27me3	SRR6307192	SRR6307193
Cercariae	SAMN02404745	SmBRE	Mixed	H3K4me3	SRX414495	SRX424005
	SAMN02404745	SmBRE	Mixed	H3K27me3	SRX395498	SRX423996
Schistosomula	SAMN03892083	SmBRE	Mixed	H3K4me3	SRX1113460	SRX1113460
	SAMN03892083	SmBRE	Mixed	H3K27me3	SRX1113461	SRX1113461
Adult	SAMN02404746	SmBRE	Mixed	H3K4me3	SRX426380	SRX443791
	SAMN02404746	SmBRE	Mixed	H3K27me3	SRX426328	SRX441110

**Table 9:** Details for the female ChIP-Seq reads available at the NCBI-SRA.

*Library*	*Biosample*	*Strain*	*Sex*	*Antibody*	*Replicate 1*	*Replicate 2*	*Replicate 3*
Cercariae	SAMN04115511	SmBRE	Female	H3K4me3	SRX1592107	SRX1592110	SRX1592114
	SAMN04115511	SmBRE	Female	H3K27me3	SRX1592106	SRX1592109	SRX1592113
	SAMN04115511	SmBRE	Female	H4K20me1	SRX1592108	SRX1592111	SRX1592115
Adult	SAMN04115514	SmBRE	Female	H3K4me3	SRX1592134	SRX1592139	SRX1592144
	SAMN04115514	SmBRE	Female	H3K27me3	SRX1592133	SRX1592138	SRX1592143
	SAMN04115514	SmBRE	Female	H4K20me1	SRX1592135	SRX1592140	SRX1592145

## Supporting information

S1 Fig **Aligned read count over all annotated TSS in the chromosome level assembled *S*. *mansoni* genome for (A) H3K4me3 and (B) H3K27me3.** Every line corresponds to an individual TSS, different colors denote differential stability of the mark during the 5 life cycle stages. Greyscale for RPKM.
(TIF)

S2 Fig Example of a long region where differences in enrichment in H3K27me3 (red) and H4K20me1 (green) can been seen in all three cercarial replicates and is absent of the three adult replicates. Differences in these regions for both marks, identified by chromstaR, are within the dotted lines.
(JPEG)

S1 File A simplified BED file with TSS and transcription end sites (TES) was generated for contigs assembled at chromosome level. Chromosome names were changed to Chr1-W.
(BED)

S2 File Description (genomic position and annotation) of all punctual (>10kb long) differences found between cercariae and adults for H3K4me3, H3K27me3 and H4K20me1. (XLSX)

S3 File Bed files with genomic coordinates of long (10–100 kb) differences found between cercariae and adults for H3K4me3, H3K27me3 and H4K20me1. (ZIP)

S4 File List of genes present in long (10–100 kb) differences found between cercariae and adults for H3K4me3, H3K27me3 and H4K20me1. (ZIP)
