# Supplementary figures and images for: Histone methylation changes are required for life cycle progression in the human parasite Schistosoma mansoni
Source: PLoS Pathog. 2018 May 21;14(5):e1007066. doi: 10.1371/journal.ppat.1007066 (PMC5983875; doi:10.1371/journal.ppat.1007066)

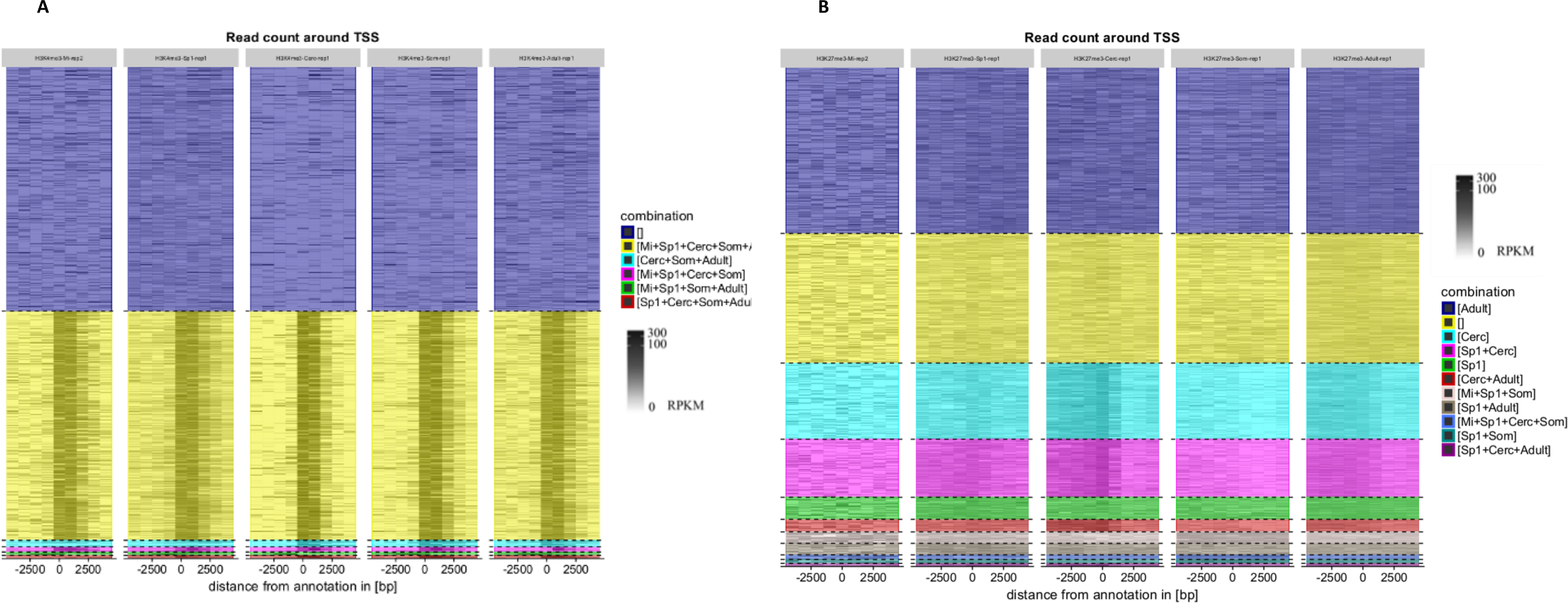

Supplement: S1 Fig — Aligned read count over all annotated TSS in the chromosome level assembled S. mansoni genome for (A) H3K4me3 and (B) H3K27me3. Every line corresponds to an individual TSS, different colors denote differential stability of the mark during the 5 life cycle stages. Greyscale for RPKM. (TIF) [file ppat.1007066.s001.tif]

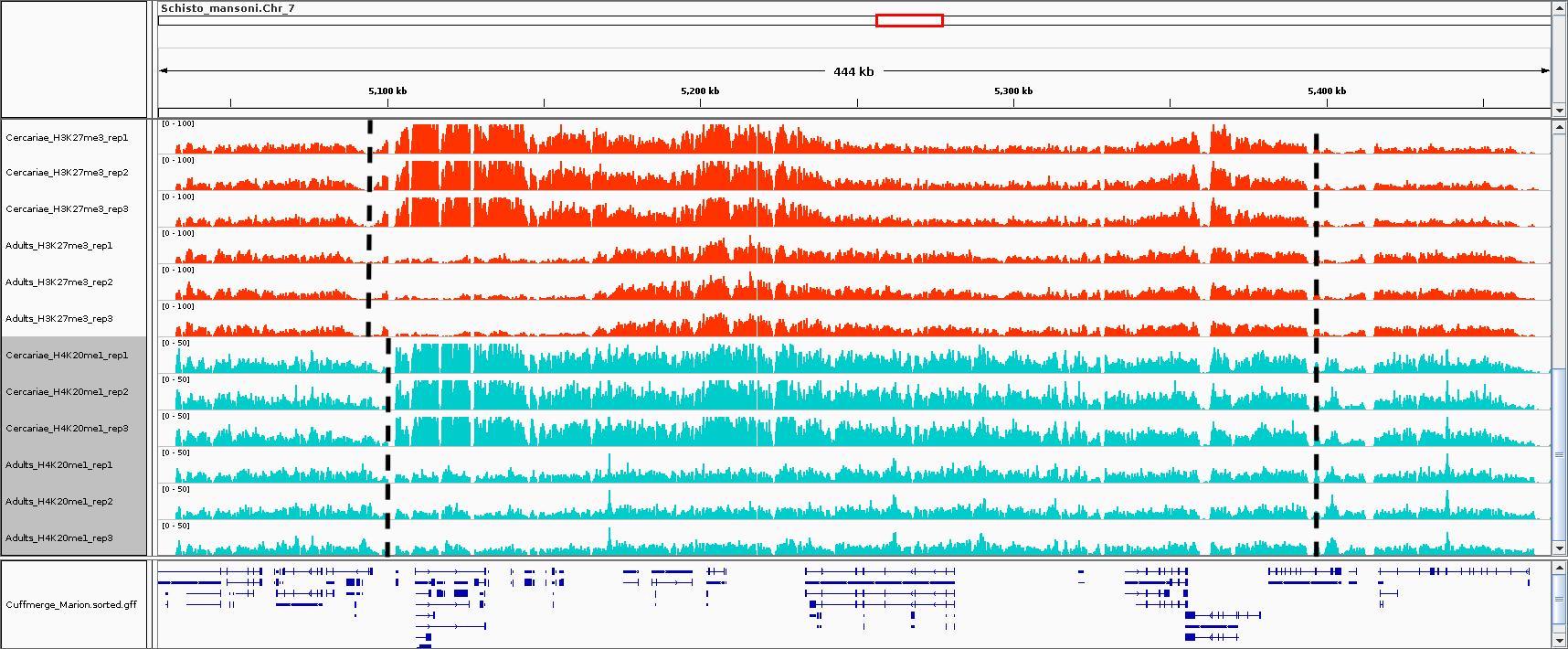

Supplement: S2 Fig — Differences in these regions for both marks, identified by chromstaR, are within the dotted lines. (JPEG) [file ppat.1007066.s002.jpeg]
